# Olive Oil Oleogel Formulation Using Wax Esters Derived from Soybean Fatty Acid Distillate

**DOI:** 10.3390/biom10010106

**Published:** 2020-01-08

**Authors:** Aikaterini Papadaki, Nikolaos Kopsahelis, Denise M. G. Freire, Ioanna Mandala, Apostolis A. Koutinas

**Affiliations:** 1 Department of Food Science Human Nutrition, Agricultural University of Athens, Iera Odos 75, 11855 Athens, Greece; imandala@aua.gr; 2 Department of Food Science and Technology, Ionian University, 28100 Argostoli, Greece; kopsahelis@upatras.gr; 3 Biochemistry Department, Chemistry Institute, Federal University of Rio de Janeiro, Technology Center, A, Lab 549, Rio de Janeiro 21941-901, Brazil

**Keywords:** structured lipids, organogel, fat replacer, wax esters, enzymatic synthesis, food processing, soybean fatty acid distillate, bioeconomy

## Abstract

Oleogelation is an emerging technology to structure oils, which can be widely used to substitute saturated and trans fats. Extra virgin olive oil is widely recognized for its high nutritional value, but its utilization in oleogel production is currently limited. In this study, extra virgin olive oil was utilized for the production of a novel oleogel using wax esters derived from soybean fatty acid distillate (SFAD), a byproduct of industrial soybean oil refining. Different concentrations (7%, 10%, 20%, *w*/*w*) of SFAD-wax esters were used to evaluate the minimum concentration requirement to achieve oleogelation. Analyses of the mechanical properties of oleogel showed a firmness of 3.8 N, which was then reduced to around 2.1–2.5 N during a storage period of 30 days at 4 °C. Rheological analysis demonstrated that *G*′ is higher than *G*″ at 20–27 °C, which confirms the solid properties of the oleogel at this temperature range. Results showed that SFAD was successfully utilized for the oleogelation of olive oil, resulting in a novel oleogel with desirable properties for food applications. This study showed that industrial fatty side streams could be reused for the production of value-added oleogels with novel food applications.

## 1. Introduction

In recent years, a societal challenge related to obesity and cardiovascular diseases, as a result of high saturated and trans fats intake, has been appraised and poses a detrimental effect not only to health but also to the healthcare system. A study in six European Union countries demonstrated that the healthcare cost due to cardiovascular diseases was over €102 billion in 2014 and it is estimated to reach up to €122.6 billion by 2020 [1]. According to [2], the reduction of saturated fat contributes to lower risk of developing such chronic diseases. In this context, the US Food and Drug Administration (US F.D.A) recently banned the use of partially hydrogenated oils in food production and set 1 January 2020 as the final compliance date, as the hydrogenation process is responsible for the formation of trans fats [3].

Oleogels have recently gained research attention, since they can serve as a healthier alternative of saturated and trans fats in several food products, including bakery, cheese, meat, and confectionery products [4,5]. Some disadvantages which characterize the oleogels are the limited types of food-grade oleogelators, the heating preparation which may result in oil oxidation, their poor compatibility with other food ingredients and their inability to deliver hydrophilic compounds [5]. On the other hand, oleogels present many technological advantages in food production. In particular, the incorporation of oleogels in food formulations is mainly intended to decrease phase separation and migration of oil phase, hence their solid-like behavior provides desirable structural characteristics to the food products applied, avoiding the usage of high amounts of saturated fats [5]. Furthermore, oleogels can be also utilized as carrier materials of bioactive compounds with the aim to improve their functional properties and control their release [6].

The oleogelation process has been recognized as an emerging technology, which has been fairly studied using plant wax esters, such as beeswax, carnauba wax, candelilla wax, sunflower wax, and rice bran wax among others [7]. Generally, waxes have been characterized as the most efficient oleogelators, among other compounds (e.g., monoglycerides, fatty alcohols, phytosterols etc.) due to their high oil-binding capacity, strong self-assembled network formation, and excellent crystallization ability [8]. Many different vegetable oils, such as sunflower oil, soybean oil, corn oil, canola oil, hazelnut oil, rice bran oil, grape seed oil, rapeseed oil, and flaxseed oil have been utilized for oleogel production with the aim to investigate their properties and their potential applications [8]. However, there are only a few studies using olive oil as the only base oil for wax-based oleogel production [9,10,11,12,13,14], whereas the most recent reports have formulated oleogels with a mixture of vegetable oils, including olive oil [15,16,17]. Olive oil oleogels have been formulated to date with the utilization of rice bran wax, carnauba wax, beeswax, and sunflower wax, whereas their physical properties point out their potential application in spreadable fat products, such as breakfast margarine [9,10,11,12]. Particularly, a sensory evaluation study of these olive oil oleogels demonstrated that more than 40% of the consumers would “definitely buy” them as a spreadable fat alternative [13]. These results highlight the necessity for further research, regarding optimization of oleogel formulation, in order to fulfill consumers’ acceptance criteria.

Enzymatically synthesized wax esters, derived from agro-industrial renewable resources, could be an alternative to plant wax esters. For instance, fatty acid distillates derived from the vegetable oil refining process could be used as substrate for the enzymatic synthesis of wax esters. The successful production of wax esters via enzymatic catalysis, using palm fatty distillates, soybean fatty acid distillate (SFAD), and microbial oil have been reported in previous studies [14,18,19], with the aim to valorize food industry byproducts within the frame of Circular Economy. In our previous studies, SFAD wax esters and microbial oil wax esters have been utilized as oleogelators in the formulation of soybean oil and microbial oil oleogels [14,20].

Therefore, the aim of this study was the formulation of a novel olive oil oleogel, through the valorization of SFAD. Specifically, SFAD wax esters were evaluated as oleogelators for the formulation of an extra virgin olive oil oleogel. Literature reports dealing with formulation of olive oil oleogels are exclusively limited in utilization of plant wax esters, whilst enzymatically synthesized wax esters for the development of novel oleogels are scarcely found in the literature. This study investigated the perspective of an olive oil oleogel formulation along with the valorization of fatty industrial byproduct streams derived from the soybean oil refining process. The evaluation of oleogel, regarding its physical properties, showed that it could be potentially applied in the manufacturing of food products. To the best of our knowledge, this is the first report proposing valorization of fatty industrial byproducts for the production of novel olive oil oleogels with potential applications in healthier food product development.

## 2. Materials and Methods

### 2.1. Raw Materials

Extra virgin olive oil was purchased from the local food market (Athens, Greece). SFAD was kindly provided by Miracema-Nuodex Chemical Industry Ltd. (Campinas—São Paulo, Brazil). Cetyl alcohol was purchased from Sigma-Aldrich (St. Louis, MO, USA) and was used for enzymatic synthesis of cetyl wax esters.

### 2.2. Enzymatic Synthesis of Wax Esters

Wax esters were enzymatically produced from SFAD and cetyl alcohol and were utilized as oleogelators. Enzymatic conversion of SFAD was carried out as described in our previous study [18,19]. Briefly, cetyl alcohol (≥95% purity, Sigma-Aldrich, St. Louis, MO, USA), which has been included in the list of food additives by the U.S. Food and Drug Administration (FDA), was utilized with SFAD at a molar ratio of 1:1. Enzymatic synthesis was performed in a solvent-free system under agitation and the reaction was initiated by addition of the commercial lipase Novozyme 435 (Sigma-Aldrich, St. Louis, MO, USA).

### 2.3. Oleogel Preparation

Olive oil was used as base oil for the production of oleogels with different concentrations (7%, 10%, and 20%, *w*/*w*) of SFAD cetyl wax esters. The base oils and cetyl wax esters were precisely weighed and the mixture was heated at 90 °C under agitation for 10 min until all the components melted. Oleogels were transferred into screw capped glass vials and cooled at room temperature for 24 h to allow gel formation. The samples were then stored at 4 °C for 30 days for texture analysis. All results reported for oleogels represent the mean values of triplicates.

### 2.4. Analytical Methods

#### 2.4.1. Crystal Morphology

The crystal morphology of wax esters and oleogels was studied using a polarized light microscope (Axiolab, Zeiss, Oberkochen, Germany) equipped with a digital camera (DSC-575, Sony, Tokyo, Japan). Freshly prepared oleogels were spread on a glass slide covered with a cover glass. Images were captured with the camera at a magnification of ×10.

#### 2.4.2. Color Analysis

A colorimeter (ChromaMeter CR-400/410, Konica Minolta, Tokyo, Japan) was employed for color analysis of the oleogel. The calibration of the colorimeter was performed with a white and a black plate. The samples were poured in cylindrical tubes with 2 cm diameter and 1 cm height. The color was recorded using CIE-L*a*b* uniform color space (CIE-Lab). Additionally, Hue angle (h*) and Chroma (C*) were evaluated using the respective equations:

(1) $$h^{*}={tan}^{-1}\left(\frac{b*}{\alpha *}\right),$$

(2) $$C^{*}=\sqrt{\alpha^{*2}+b^{*2}}.$$

#### 2.4.3. Rheological Analysis

A Discovery HR 3 Hybrid Rheometer (TA Instruments, New Castle, DE, USA) equipped with a parallel plate geometry system and a measuring gap of 700 μm was utilized for the rheological properties of the oleogel. Samples were transferred from the storage temperature (4 °C) onto the Peltier plate preheated at 20 °C and held for 5 min. The parameters of viscosity, storage modulus (*G*′), loss modulus (*G*″), and loss tangent (tan *δ* = *G*″/*G*′) versus temperature were determined during a heating cycle up to 80 °C, ramped at a rate of 2 °C/min and a steady shear rate of 100/s.

#### 2.4.4. Texture Analysis

A texture analyzer (Instron 1011, Norwood, MA, USA), equipped with a 50-N load cell, was employed for the determination of texture of the oleogel during a storage period of 1–30 days. Glass tubes with internal diameter of 2 cm were filled with 15 mL of oleogel and stored at 4 °C. The penetration force was measured at 2 cm depth of samples after plunging a cylindrical probe with a penetration speed of 100 mm/min. The maximum force was reported as a penetration force (N).

#### 2.4.5. Melting Behavior

A differential scanning calorimetry (DSC) analysis (Q100 model, TA Instruments, New Castle, DE, USA) was employed to determine the thermal properties of oleogels. Around 5–10 mg of samples was precisely weighed and hermetically sealed in an aluminum pan. The samples were heated to 140 °C at a 10 °C/min heating rate, followed by cooling at −20 °C at a 10 °C/min heating rate and then reheated to 140 °C at a rate of 5 °C/min [13]. An empty pan was used as reference.

### 2.5. Statistical Analysis

The statistical differences among treatments were estimated by analysis of variance (ANOVA). Whenever ANOVA indicated a significant difference between variables at a significance level of 5% (*p* < 0.05), the Tukey’s HSD (honest significant difference) test was carried out using the Microsoft Excel software.

## 3. Results and Discussion

### 3.1. Olive Oil Oleogel Production

Wax esters were produced, using cetyl alcohol and SFAD, via enzymatic catalysis. The details of the protocol have been previously reported, which results in high SFAD to wax ester conversion yields [18,19]. Olive oil oleogels were prepared using SFAD wax esters at the concentration of 7%, 10%, and 20% (*w*/*w*). Results showed that a minimum quantity of 20% (*w*/*w*) was required to achieve oleogelation. The concentrations of 7% and 10% resulted in a weak oleogel network that was flowing on an inclined surface. For this reason, olive oil oleogels with 20% of SFAD wax esters were utilized for the subsequent characterization of their physical properties.

### 3.2. Microstructure Analysis

**Figure 1:**
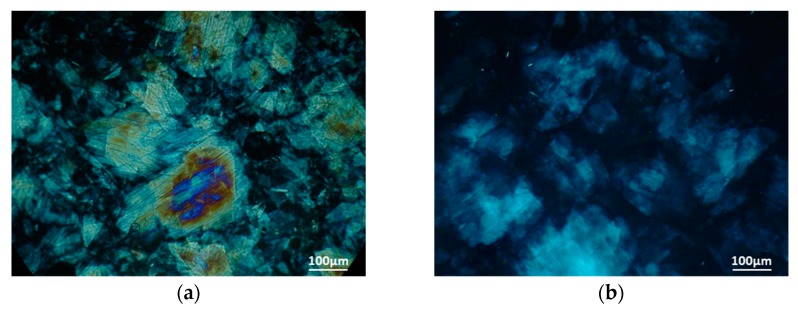
Polarized light microscopy micrographs of (**a**) wax esters derived from soybean fatty acid distillate (SFAD wax) and (**b**) oleogel produced from extra virgin olive oil and SFAD wax.

Crystal morphology, for both SFAD wax esters and oleogel, was evaluated using a polarized light microscope, which demonstrated the formation of large flake-shaped crystals with length higher than 100 μm (Figure 1a,b). Similar crystal formation was also determined for oleogels formulated by soybean oil and SFAD wax esters [20]. Generally, the shape and size of crystals are affected mainly by the type of oleogelator. Τhe use of plant waxes, such as beeswax, carnauba wax, rice bran wax, and sunflower wax, have resulted in the formation of needle-like crystals up to 50 μm in the case of rice bran wax, whereas carnauba wax and candelilla wax formed spherulitic structures (less than 10 μm) in olive oil oleogels [9,12,13].

### 3.3. Color and Melting Temperatures Analysis

Table 1 depicts the results concerning the color analysis. Positive b* values indicates the yellowish color, whereas the negative values of a* indicate the tendency for greenish shades of the produced oleogels. The h* value refers to the hue’s location in the CIE-L*C*h color range, where red is expressed by 0°, yellow by 60°, green by 120°, and blue by −120°. Olive oil oleogel presented high h* values (79.5°) due to its high intensity to yellowish color. Similar color ranges to this study have been reported in olive oil oleogels prepared with plant waxes, such as sunflower wax (a* = −7.95; b* = 36.12) and beeswax (a* = −6.01; b* = 19.87) [12]. L*, a*, and b* values were also found similar to soybean oil oleogels with SFAD wax (27.1, 0.1, 15.5, respectively) [20]. The color of the oleogel produced in this study is close to commercial structured lipids, such as breakfast margarine (L* = 85.34, a* = −2.68, and b* = 12.17) [12]. It should be certainly taken into consideration that breakfast margarine also contains many other different ingredients, such as emulsifiers, which alters the color and other properties. However, a potential application of this olive oil oleogel, as fat substitute in commercial spreadable fat products, will provide a familiar color appearance to the final product, which will enhance its acceptance by the consumers.

**Figure 2:**
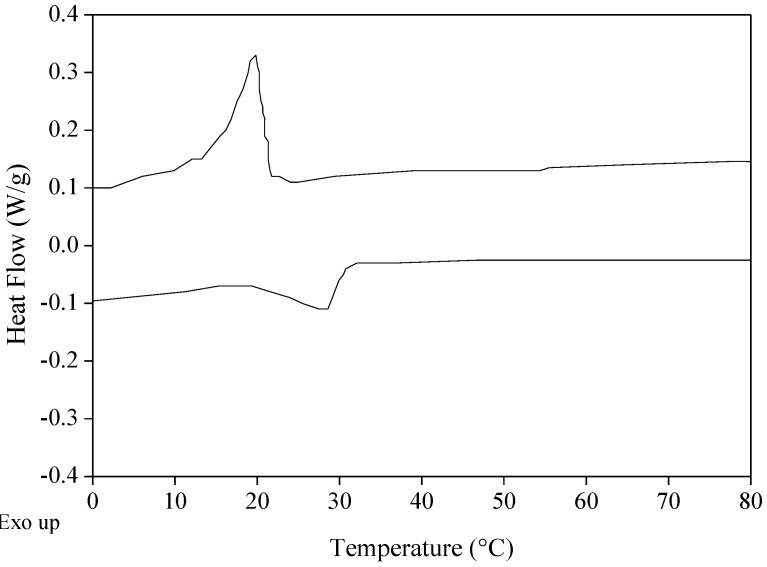
Differential scanning calorimetry (DSC) thermogram of the oleogel produced from extra virgin olive oil and wax esters derived from soybean fatty acid distillate (SFAD wax).

DSC analysis showed a peak melting temperature at 28.5 °C and a complete melt of all the components at 29.8 °C (Table 1, Figure 2). The melting temperatures of olive oil and SFAD wax esters are –3.96 °C and 43.8 °C, respectively [12,18]. Obviously, the melting temperature of the oleogel was affected by the melting point of the oleogelator. Yilmaz and Öğütcü [12,13] have reported a peak melting temperature around 44–50 °C for olive oil beeswax oleogels and 58–63 °C for olive oil sunflower oleogels, which were strongly dependent on the melting points of beeswax (63.15 °C) and sunflower wax (76.29 °C), respectively. Likewise, olive oil carnauba wax oleogels had a melting temperature of 65–76 °C [10]. Similar melting temperatures to this study have been demonstrated for soybean oil and SFAD wax esters (around 30–32 °C) in a previous study [20]. The melting profile of the produced oleogel indicates that it could be used in food applications, such as confectionery products, at which a melting temperature of less than 35 °C is required [21].

### 3.4. Rheological Properties

**Figure 3:**
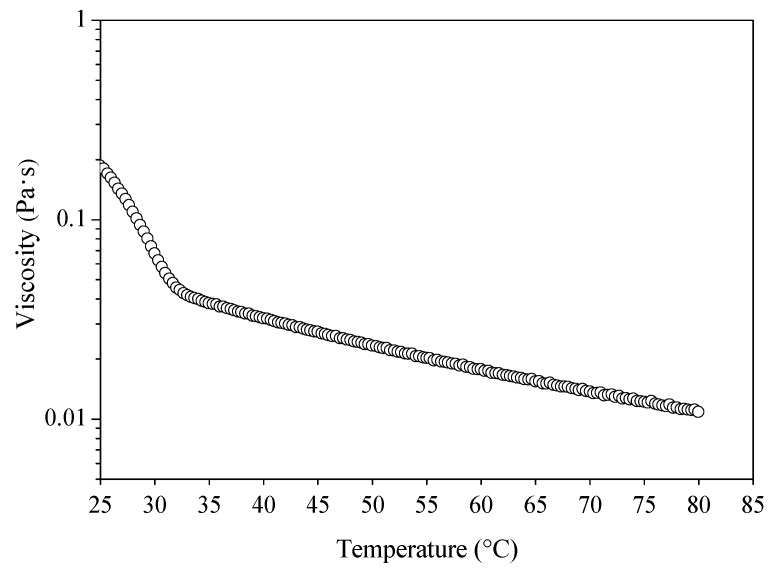
Evaluation of viscosity versus temperature of the oleogel produced from extra virgin olive oil and wax esters derived from soybean fatty acid distillate (SFAD wax).

Figure 3 presents the changes of the viscosity versus temperature. A heating cycle was performed from 25 to 80 °C, at which a transition from the solid-state to a viscous-state was observed by the change of the slope at around 31 °C. Similarly, oleogel made from soybean oil and SFAD wax presented an identical shift at around 32 °C [20]. The change of slope is related to the melting temperatures of the oleogel [14,22] and depicts the changes of the crystal network as a result of the increased temperature.

**Figure 4:**
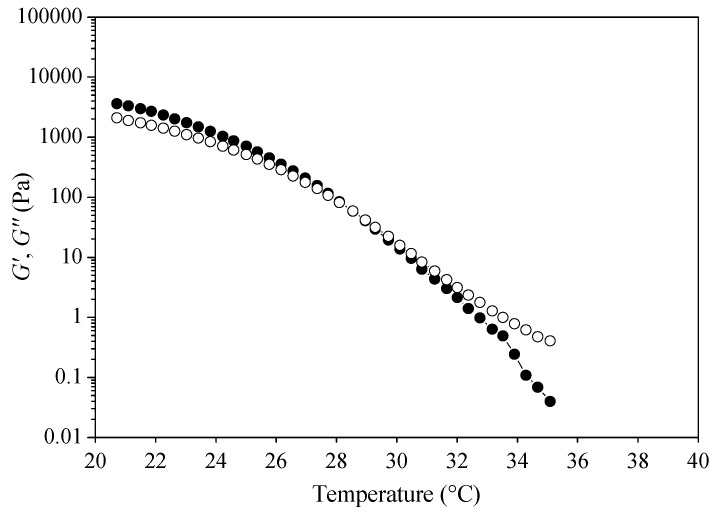
Evaluation of storage modulus (*G*′) and loss modulus (*G*″) versus temperature of the oleogel produced from extra virgin olive oil and wax esters derived from soybean fatty acid distillate (SFAD wax).

As seen in Figure 4, oleogel presented a *G*′ value of 3.6 × 10^3^ Pa, which is an indicator of the gel strength. Moreover, *G*′ was higher than *G*″ (*G*′ *> G*″) at the temperature range of 20–27 °C demonstrating that the oleogel presented more solid-like properties at these temperatures. A cross over point (*G*′ = *G*″), which depicts the transformation of the gel into a sol, was observed at 28–29 °C. These results are also in agreement with the melting temperatures from DSC analysis. Tan *δ* values ranged from 0.6 to 0.9 within the temperature range of 20–28 °C, suggesting that oleogels behaved more like a solid at these temperature values. It has been suggested that a low tan *δ* value indicates a more stable crystal network structure [23], whereas values less than 0.1 (tan *δ* ≤ 0.1) indicate a true gel. Rheological analysis of olive oil oleogels are scarcely found in the literature. Our previous study in olive oil oleogel preparation with microbial oil derived wax esters presented a higher *G*’ value (around 10^4^ Pa) [14] and higher cross over point (33.7 °C) than this study. Obviously, the use of different oleogelators affects the oil-oleogelator interaction and thus the crystal network formation and its rheological behavior.

### 3.5. Textural Properties

**Figure 5:**
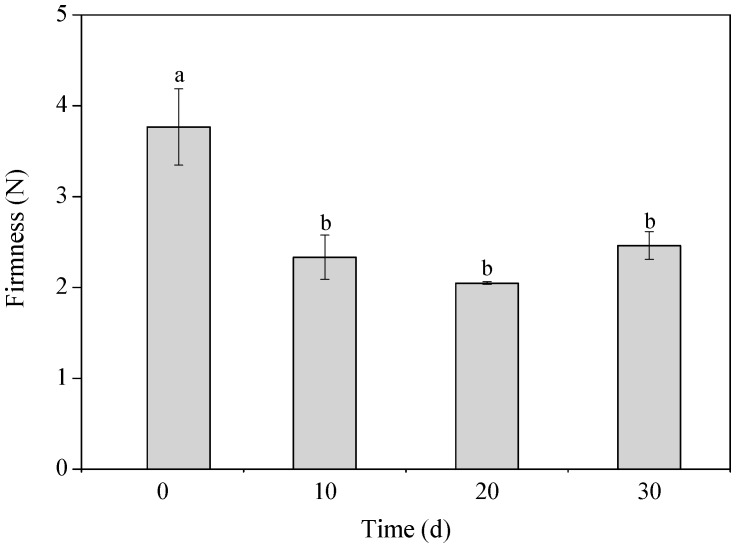
Texture analysis of the oleogel produced from extra virgin olive oil and wax esters derived from soybean fatty acid distillate (SFAD wax), during a storage period of 1–30 days at 4 °C. Different letters indicate significant differences (*p* < 0.05).

Results concerning the firmness of the oleogel are presented in Figure 5. The highest firmness of 3.8 N was determined at the first day of storage. During a storage period of 30 days the firmness was significantly decreased (*p* < 0.05), but it remained stable (2.1–2.5 N) until the 30th day (*p* > 0.05). Higher firmness levels have been obtained in previous studies for olive oil oleogels with 10% carnauba wax (5.4 N, 4 °C, day 0) [10] and with 20% microbial oil derived wax esters (6 N, 4 °C, day 1) [14]. The low firmness level of olive oil oleogel could be explained by the formation of large crystals, as depicted in SEM, due to the less effective surface area and the fewer contact points between the large crystal particles [24]. Texture is an important parameter for consumers’ acceptance and is conventionally regulated using hydrogenated oils. Generally, spreadable products, such as breakfast margarine has a firmness of 4 N at 4 °C [10]. In our case, olive oil oleogel could be potentially applied as a fat substitute in a mixture with other oleogels, hardfats, or even using a combination of high and low melting waxes [25] with the aim to provide appropriate levels of firmness to the final product.

Beside the wide utilization of oleogels as fat substitutes, they can also be used as carrier materials of bioactive compounds. Several preliminary studies have reported the utilization of gels as carrier materials of carotenoids, polyphenols, flavonoids, omega-3 fatty acids, probiotics, and enzymes [6,20,26,27,28,29], targeting improved properties and enhanced functionality of the respective bioactive compounds. Thus, further studies should be focused on both physical and functional properties of the produced oleogels, which will lead to successful market applications.

## 4. Conclusions

This study presented the development of a novel oleogel made from olive oil and wax esters derived from SFAD. Olive oil oleogel presented solid properties (*G*′ > *G*″) at room temperature and demonstrated desirable melting profile for food applications. Its firmness level was found relatively low as compared with other olive oil oleogels. Nevertheless, further investigation in oleogel production using a mixture of enzymatically prepared wax esters and high melting plant waxes could result in enhanced properties. This study highlights that the enzymatic conversion of fatty industrial byproduct streams into wax esters and their further application in oleogel production could be an alternative route for strengthening bioeconomy development.

## Figures and Tables

**Table 1:** Evaluation of color and melting temperature of the oleogel produced from extra virgin olive oil and wax esters derived from soybean fatty acid distillate (SFAD wax).

Parameters		Measured Value
Color	L*	25.7 ± 0.0
	a*	−2.9 ± 0.1
	b*	15.4 ± 1.1
	C*	15.71
	h*	79.5°
Melting temperatures (°C) ^1^	*T_on_*	23.2 ± 1.3
	*T_p_*	28.5 ± 1.1
	*T_com_*	29.8 ± 0.9

^1^ Melting temperatures corresponds to *T_on_*: onset melting temperature; *T_p_*: maximum peak temperature; *T_com_*: completion of melting.
